# Towards Understanding Long COVID: SARS-CoV-2 Strikes the Host Cell Nucleus

**DOI:** 10.3390/pathogens12060806

**Published:** 2023-06-06

**Authors:** Laura Lafon-Hughes

**Affiliations:** 1 Departamento de Genética, Instituto de Investigaciones Biológicas Clemente Estable, Ministerio de Educación y Cultura, Montevideo 11600, Uruguay; lauralafon2010@gmail.com; Tel.: +598-2-93779096; 2 Grupo de Biofisicoquímica, Departamento de Ciencias Biológicas, Centro Universitario Regional Litoral Norte, Universidad de la República (CENUR-UdelaR), Salto 50000, Uruguay

**Keywords:** SARS-CoV-2, nucleocytoplasmic shuttling, NLS, spike, Nucleocapside, Nsp, ORF9a, accessory proteins, sgRNA, retrotranscription

## Abstract

Despite what its name suggests, the effects of the COVID-19 pandemic causative agent “Severe Acute Respiratory Syndrome Coronavirus-2” (SARS-CoV-2) were not always confined, neither temporarily (being long-term rather than acute, referred to as Long COVID) nor spatially (affecting several body systems). Moreover, the in-depth study of this ss(+) RNA virus is defying the established scheme according to which it just had a lytic cycle taking place confined to cell membranes and the cytoplasm, leaving the nucleus basically “untouched”. Cumulative evidence shows that SARS-CoV-2 components disturb the transport of certain proteins through the nuclear pores. Some SARS-CoV-2 structural proteins such as Spike (S) and Nucleocapsid (N), most non-structural proteins (remarkably, Nsp1 and Nsp3), as well as some accessory proteins (ORF3d, ORF6, ORF9a) can reach the nucleoplasm either due to their nuclear localization signals (NLS) or taking a shuttle with other proteins. A percentage of SARS-CoV-2 RNA can also reach the nucleoplasm. Remarkably, controversy has recently been raised by proving that-at least under certain conditions-, SARS-CoV-2 sequences can be retrotranscribed and inserted as DNA in the host genome, giving rise to chimeric genes. In turn, the expression of viral-host chimeric proteins could potentially create neo-antigens, activate autoimmunity and promote a chronic pro-inflammatory state.

## 1. Introduction

Severe Acute Respiratory Syndrome Coronavirus 2 (SARS-CoV-2) is a coronavirus that caused the COVID-19 pandemic. COVID-19 was initially considered an acute respiratory disease. Unsurprisingly, it turned out to be a disease that can affect several body organs or turn systemic, and an important percentage of the population is currently coping with Long COVID.

The extent of the consequences related to COVID has prompted financial support for research and is allowing us to expand our knowledge on these ss(+) RNA viruses. The initial scheme of the viral cycle, which excluded the host cell nucleus, is being enriched. Here, we revise the SARS-CoV-2 infection from the nuclear side of the cell as well as the main hypotheses to explain Long COVID.

### 1.1. Coronaviruses Structure, Translation, Replication and Transcription

Coronaviruses (Coronavirinae subfamily, Coronaviridae Family, Nidovirales order) are enveloped viruses harboring single-stranded positive RNA [ss (+) RNA] that cause respiratory, enteric, hepatic and neurological diseases in multiple species, including several mammals and birds. Four genera are recognized (α, β, γ, δ), but only two of them (α, β) are known to cause zoonotic infections in humans.

Severe Acute Respiratory Syndrome Coronavirus 2 (SARS-CoV-2) belongs to β genera, together with Bovine Coronavirus (B-CoV), Canine Respiratory Coronavirus (CR-CoV), Human Coronavirus OC43 (H-CoV-OC43), Mouse Hepatitis Virus (MHV), Porcine Hemagglutinating Encephalomyelitis Virus (HEV), Rat Coronavirus (RCV), human HCoV-HKU1, Severe Acute Respiratory Syndrome Coronavirus (SARS-CoV) and Middle East Respiratory Syndrome Coronavirus (MERS-CoV) [1].

**Figure 1:**
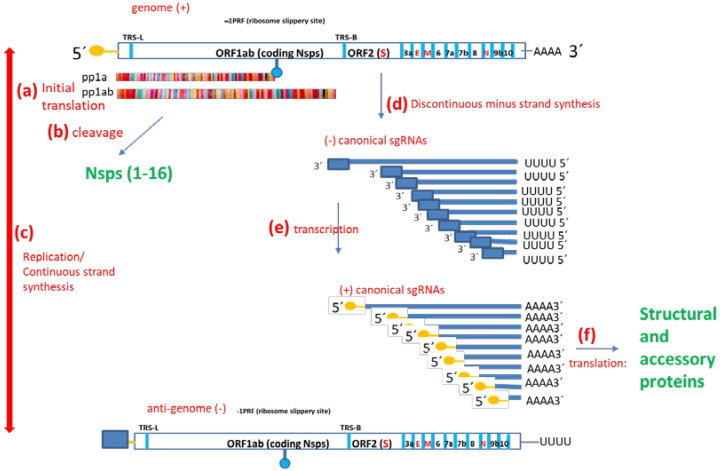
SAR-CoV-2 RNA and protein synthesis. SARS-CoV-2 genome is ss+RNA. Upon cell invasion, (a) two polypeptides are synthesized (pp1a and pp1ab) and then (b) cleaved to obtain Nsps 1-16. Then, (c) replication originates de antigenome and vice versa; the antigenome is the template to synthesize more copies of the genome. The proportion is about 1 antigenome: 350 genomes. (d) Discontinuous RNA synthesis gives rise to (−) canonical subgenomic, which are then (e) transcribed to (+) sgRNAs, the messengers that are (f) translated to obtain the structural and accessory proteins. Hundreds of different non-canonical sgRNAs do also co-exist.

SARS-CoV-2 genomic structure, transcription, translation and replication (Figure 1) follows Coronavirus (CoV) rules. CoV ss(+) genomic RNA (gRNA) serves as a template for both translation (as an mRNA) and replication. The coronavirus genome is organized in such a way that the proteins that will be needed first are translated first. CoV genomes comprise at least five ORFs [2]. ORF1ab covers two-thirds of the genome and codes for 16 non-structural proteins (Nsps), which are identified with a number according to the order of appearance in the genome (from 5′ to 3′). ORF2 codes the Spike protein (S). The other ORFs code some small proteins called “accessory proteins” as well as the Envelope protein (E), Membrane protein (M) and Nucleoprotein (N). Translation generates an intermediate product called polypeptide 1a (pp1a) or the longer pp1ab, depending on whether the ORF1a termination codon is translated as such or bypassed/avoided through a slippery ribosome mechanism called −1 programmed ribosomal frameshifting (−1 PRF) [3,4,5]. In any case, Nsps are obtained after proteolytic cleavage at specific sites through the action of two proteases which are also coded within the polypeptide, namely Nsp3 or papain-like protease (PLPRO) and Nsp5 or 3-chemotrypsin-like protease (3-CLPRO) [6]. Importantly, Nsp1 codes a host mRNA translation inhibitor and pp1ab (but not pp1a) harbors Nsp12, the main RNA-dependent RNA-polymerase (RDRP). Replication generates the antigenome or complementary viral sequence (−RNA). In turn, the antigenome is the transcription template [7]. There is a single leader transcription regulatory sequence (TRS-L) upstream ORF1ab. Moreover, body transcription regulatory sequences (TRS-B) are located upstream of other ORFs. Both continuous and discontinuous transcription can occur. Continuous transcription is a canonical process that consists of using the antigenome (−RNA) as a template to obtain new copies of the +RNA. In contrast, discontinuous transcription involves template “pseudocircularization”, allowing a template switch and the acquiring of nested shorter mRNAs called subgenomic mRNAs (sgRNAs). Except for Nsps, translated from gRNA, all the other proteins, including the components of the spike trimeric complex (S), envelope (E), matrix (M) and nucleocapsid (N), are translated from sgRNAs. Interestingly, the phosphorylation of the viral N protein by a host cell kinase is involved in the switch from discontinuous to continuous transcription.

### 1.2. Coronavirus Acute vs. Persistent Infection and Chronic Effects

According to a study carried out more than three decades ago by Kristensson and Norrby [8], seven families of RNA viruses do somehow produce persistent infections in the mammalian central nervous system, which correlate with signs of disease such as neurotransmission alterations, progressive cellular destruction or demyelination.

Coronaviruses induce persistent infections in bats, cats, mice and humans. Theoretically, viral persistence in a population can be achieved in two ways: first, if each individual is infected and then spreads the infection and subsequently recovers (thus, the virus persists in the population but not in each individual); second, through a persistent or chronic infection in each individual. Little brown bats in North America in captivity can harbor the coronavirus in their intestines and lungs over a 4-month hibernation period without showing pathological signs. Nevertheless, under a co-infection with the white-nose syndrome fungus, intestinal coronavirus production increases 60× [9]. Feline infectious peritonitis is a highly lethal coronavirus infection. It behaves as an intermittent infection, with disease episodes involving enhanced viral replication and short periods of apparent convalescence. Each wave of viral replication induces an antiviral T-cell response and is coincident with fever, weight loss, and T-cell depletion [10]. In mice, MHV can produce both acute and chronic diseases. Infection after intracerebral inoculation is normally fatal, but survivors exhibit oligodendrocyte infection and demyelination. Neutralizing antibodies protect from death; instead of fatal encephalomyelitis, demyelination occurs. The outcome of intracerebrally inoculated MHV can be a biphasic disease with hepatitis and then a chronic disease with demyelination. The virus in the persistent infection can affect the *substancia nigra* and favor Parkinsonism. Moreover, intranasal inoculated MHV enters the central nervous system through the olfactory and trigeminal nerves, with surgical ablation of the olfactory pathway protecting from the infection [11].

During the 2003 SARS outbreak, it was soon demonstrated that, in contrast to the lytic infection observed in VERO green monkey kidney cells, SARS produces a persistent infection in the human colorectal adenocarcinoma-derived LoVo cell line [12]. Persistent infection by HCoV-229E and HCoV-043 had also been reported by that time in human cell lines derived from oligodendroglioma (MO 3.13), human neuroglioma (H4) and human malignant glioma (U-87 MG, U-373 MG). A case of a child with acute disseminated encephalomyelitis associated with HCoV was also published as a warning, highlighting that HCoV might behave as MHV [13]. At the time, the accepted (WHO) clinical SARS definition required fever and one or more symptoms of lower respiratory tract illness (cough, difficulty of breathing, shortness of breath) for a potential case to be considered. Therefore, data from patients with no respiratory symptoms were not even collected initially; SARS-CoV-infected patient data were probably not appropriately collected. Nevertheless, in 2011, a case–control study described a chronic post-SARS syndrome, including widespread musculoskeletal pain, fatigue, depression, and sleep disturbances reminiscent of fibromyalgia and chronic fatigue syndrome [14]. Moreover, a study involving postmortem analysis of tissues from 10 SARS-CoV patients reported “widespread dissemination of the virus in the immune cells of the blood, spleen and lymph nodes, as well as in the epithelial cells of lungs, trachea, bronchi, distal renal tubules, mucosa and submucosa of the intestines, and neurons in the brain”. The authors consider that SARS infection is more devastating than HIV for the immune system. They also warn that, as SARS is detectable in blood, stool, intestines, and urine, body fluid or fecal transmission might be possible. The skeletal muscles are also affected in 30% of SARS patients, as evidenced by muscle weakness and serum creatine kinase (CK) levels [15]. The exocrine and endocrine pancreas [16] are also affected. Hours to days after inhalation of HCoV-OC43 or SARS-CoV, the respective CoV was detected in the olfactory bulb, pyriform cortex, brain stem and spinal cord [17]. HCoV-OC43 and HCoV—229E have been detected in the brain tissue of multiple sclerosis patients by in situ hybridization, PCR, immunohistology or virus isolation. One patient was 11 months old with severe immunodeficiency and lethal encephalitis [18].

### 1.3. Canonical SARS-CoV-2 Productive Infection Cycle

**Figure 2:**
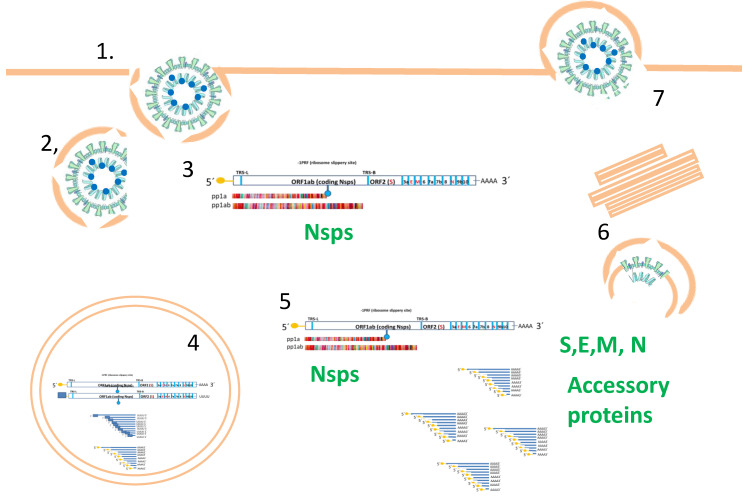
Typical SAR-CoV-2 infective cycle representation. The steps comprise the following: (1) Receptor binding, attachment and entry by endocytosis. (2) Uncoating. (3) Cytoplasmic primary translation and protease activation (ORF1a, ORF1ab; Nsps 1-16)ñ. (4) Replication and transcription in double-membrane vesicles (DMVs). (5) Additional translation of sgRNAs (coding structural and accessory proteins). (6) Virion assembly at endoplasmic reticulum Golgi intermediate compartment (ERGIC) and maturation. (7) Release by exocytosis.

The canonical life cycle of SARS-CoV-2 infection (Figure 2) comprises the following steps: (i) attachment to host cell surface and viral penetration; (ii) uncoating; (iii) primary translation, protease activation and replication-transcription complex (RTC) formation; (iv) synthesis of viral RNA; (v) translation of sgRNAs; (vi) molecular assembly and (vii) release of viral particles [19]. These steps describe a successful, productive infection, which seems to occur without a single direct interaction of viral proteins with the nuclear compartment. The structural proteins are mainly involved in viral particle assembly. Most Nsps are essential for successful viral replication [20] and have ascribed functions [19], including the inhibition of host mRNAs translation, binding to prohibitins (PHB1/2) to modulate mitochondrial functions, protease activity, viral replication-transcription, ER membrane modification and induction of double-membrane vesicles. According to this scheme, Nsps functions occur outside the nucleus. SARS-CoV-2 sgRNAs are the canonical or non-canonical products of distinct types of fusion/junction sites. Hundreds of non-canonical sgRNAs have been identified, including both in-frame and out-of-frame fusion products, with out-of-frame non-canonical sgRNAs significantly outnumbering in-frame non-canonical sgRNAs (by ~60%) [21].

Typically, the host cell nucleus is not even drawn in a SARS-CoV-2 life cycle scheme. Nevertheless, there is cumulative evidence showing that viral components interact with the host cell nucleus indirectly or directly. This review aims to argue that the interaction of SARS-CoV-2 with the nucleus is understudied and is worth studying since some clues to understanding Long COVID may be there.

## 2. Search History, Criteria and Tools

This review is not the product of a single-day search. It is instead the product of having followed the coronavirus biology since the pandemic was declared. In fact, part of the text regarding the biology of formerly known coronaviruses can be read in a preprint which, due to manuscript overload, was never evaluated nor published. At that time, the intention of the manuscript was to argue that there were good reasons to protect our young population. It was already quite clear that COVID was expected to affect several body systems, and Long COVID was foreseen (https://preprints.jmir.org/preprint/21388, accessed on 4 June 2023). During the pandemic, the author maintained fluent communication with several researchers as part of the Grupo Uruguayo Interdisciplinario de Análisis de Datos de COVID-19 (GUIAD-COVID19). In this group, she did not perform quantitative data analysis. Instead, at that time, she was one of the actors who was constantly reading to stay up-to-date with the scientific world’s news regarding the pandemic, and she kept doing so after the pandemic was “finished”. During pandemics and probably related to vaccine acceptance, the cell nucleus was named mainly to say that it was not involved in the coronavirus cycle. However, the putative involvement of the nucleus is suspected in any long-time cellular process. This work is biased towards the nucleus on purpose since it aims to argue that the direct and indirect interaction of SARS-CoV-2 elements (proteins and RNAs) with the nucleocytoplasmic shuttling system and with nuclear components exists and deserves further research. Several simple searches matching, for example, SARS-CoV-2 or its elements with “host response”, ” host cell nucleus” or “nucleus”, were done through Google’s search engine.

Currently, we are dealing with the “yin and yang” of artificial intelligence (AI). On one side, we biologists have been painfully aware over the last decades that we were producing much more information than we could possibly manage to analyze. Thus, enhanced analysis power is welcome. On the other side, the power inherent to not only having the information but also having the means to decide how to distribute it and to whom is so near to God’s power that it generates a sense of dizziness. We can ignore what is going on or try to deal with it. For this reason, the author did her first and up-to-now single incursion in ChatGPT before starting to write this manuscript. She talked to this AI both in Spanish and English, beginning with the topic of an Uruguayan national hero called Josée Gervasio Artigas, then moving on to horticulture, then endoreduplication and finally, SARS-CoV-2 proteins in the nucleus (see Supplementary Material File S1). What she learned in so doing was the following:

(1) ChatGPT is good at writing in a structured way, such as in an essay;
(2) ChatGPT can build tables but does not fully understand the content; thus, it cannot order the items in a table according to successive months of the year;
(3) Importantly, the quality of the answer you get is strongly related to the quality of the question you ask. For example, it will give you a canonical conservative view of the genetic content of mammalian cells unless you name endoreduplication when you posit the question, or if you pose the wrong question, it will consider Macrodomain as a protein instead of a protein domain;
(4) Most citations, if asked, are wrong; thus, it is still difficult to track the information source;
(5) However, it is such a fast learner that after three interactions, this AI learned that the answers given to this author had to include citations. Amazing and scary at the same time!

In the end, some of the correctly cited research publications generated by ChatGPT were included in this review (thus, it was used as a complementary searching tool). Anyway, for the sake of curiosity and in order to slowly get to know what is going on, the whole conversation, which was copy–pasted before closing the session, is available as Supplementary Material File S1.

## 3. SARS-CoV-2 Direct and Indirect Interactions with the Host Cell Nucleus

Table 1, Table 2 and Table 3 show the involvement of SARS-CoV-2 non-structural, structural and accessory proteins (column I) in the diminution of the inflammatory/antiviral response (columns II and III), interactions with host NTRs or Nups (column IV), detection in the nucleus (column V) and highlights. Columns II and III refer to the impairment of IFN signaling. Upon viral infection, pattern recognition receptors such as Toll-like receptors (TLR) or RIG-I receptors are activated in immune cells (macrophages, monocytes, neutrophils, dendritic, epithelial) by foreign viral molecules, leading to NF-KB and IRF-dependent transcription of inflammatory genes and IFN-I/III. Then, IFNs act as autocrine or paracrine signals, leading to STAT1/2-dependent transcription of IFN-stimulated genes (ISGs). Column II is focused on the impairment of signals from receptor activation to transcription factor phosphorylation. “True” means that the viral protein inhibits the signaling cascade at a point upstream of transcription factor (TF) phosphorylation. When phosphorylation itself is inhibited, it is indicated as No: TF-P (e.g., noNF-KB-P). Once phosphorylated, the transcription factor is ready to be imported to the nucleus. Column III refers to known cases of modulation of nuclear import of these pro-inflammatory transcription factors by individual SARS-CoV proteins. Additional mechanisms of immune evasion exist and are explained in other reviews [2]. Column (IV) lists some known interactions of SARS-CoV-2 proteins with NTRs or Nups. Column (IV) states whether each NSp has ever been detected in the nucleus, either after protein transfection and overexpression [3] or in other circumstances. TLR: Toll-like receptor; IFN: interferon; ISG: interferon-stimulated genes; TF: transcription factor. NTR: nuclear transport receptor), including Importins, Exportins or bifunctional receptors); Nups: nuclear pore proteins; PAR: poly (ADP-ribose). G4s: G-quadruplexes.

### 3.1. SARS-CoV-2 Modulates IFN Signalling

Several signaling pathways activated by human coronaviruses, which modulate the antiviral immune response and contribute to the pathogenesis, had already been studied in 2019 [38] and seemed to have their counterpart in SARS-CoV-2. Upon infection, immune cells recognize foreign viral antigens and molecules (PAMPs) via pattern recognition receptors (PRRs), such as Toll-like receptors (TLRs) and RIG-I-like receptors (RLRs), thus stimulating the NF-KB and IRF3-dependent transcription of cytokines and IFNs, and subsequently inducing host immune responses. In turn, secreted IFNs bind to their cell surface receptors, activating STATs signaling to promote antiviral responses through IFN-stimulated genes (ISGs). Several SARS-CoV-2 proteins contribute to host immune escape. Nsps block both IFN synthesis and IFN-dependent transcription cascades at several points, from the PRRs to phosphorylation and/or nuclear translocation of the transcription factors NF-KB, IRF3 or STATs [22,25,39]. Nevertheless, a functional luciferase assay testing 23 SARS-CoV-2 proteins indicated that certain ones would increase IFN-β synthesis (Spike protein and Nsp2) or IFN-β-dependent transcription [39]. Interestingly, the same transcription factors (e.g., NF-KB) are involved in the regulatory sequences of an endogenous retrovirus LTR69 locus, termed Dup69, which has been activated by SARS-CoV-2 [40]. Regarding modulation of the host immune response, according to Zhao et al. [41], the SARS-CoV-2 N protein (which they did not detect inside the nucleus) has a biphasic effect on IFN-I production: while low-dose N reduces the phosphorylation and nuclear translocation of IRF3, STAT1, and STAT2, high-dose N protein has the opposite effects. NF-KB hyperactivation is a well-known mechanism to promote cytokine storms [28]. This could contribute to an explanation of how COVID-19 patients undergo insufficient IFN-I production (immunodepression) in early infection, which can be followed later by a cytokine storm (overactive immune response) [41]. Interestingly, the biphasic effect of N protein may be at least in part explained by liquid–liquid demixing phenomena, which occurs only at high N concentrations.

In turn, certain SARS-CoV-2 Nsps (NSP8 and NSP5) could be linked to oncogenic pathways [27].

### 3.2. SARS-CoV-2 Regulates Nucleocytoplasmic Shuttling

The main elements involved in nucleocytoplasmic transport are the Nucleoporins (Nups) that constitute the Nuclear Pore Complexes (NPCs), the Nuclear Transport Receptors (NTRs) which include importins, exportins and Bidirectional NTRs and RanGTP/GDP gradients. Viral hijacking of nuclear transport is known to be used by different viruses [42].

It was already known that SARS-CoV and MERS interacted with certain importins, thus blocking the nuclear translocation of NF-KB or STAT. In turn, SARS-CoV-2 interacts with Nup 37, Nup54, Nup58, Nup62, Nup88, Nup93, Nup160, Nup188, Nup210, Nup214, Nup98-RAE1, NUTF2, IPO5, IPO8, RanBP6, importin-β1, CRM1, XPOT, THOC3 and RanBP2/Nup358 [28]. SARS-CoV-2 infection reduced RanBP2/Nup358 protein. Thus, SARS-CoV-2 increased NF-KB, leading to cytokine storms [28]. Ivermectin, which reduces viral load 5000 fold, not only binds strongly to SARS-CoV-2 Spike protein glycan sites, thus diminishing their interaction with blood and epithelial cells (thus inhibiting hemagglutination induced by Spike) [43] but also destabilizes Imp1 [19].

As reviewed by Shen et al. [28], SARS-CoV-2 infection blocks the nuclear export of host antiviral mRNAs and nuclear translocation of STAT1 through the interaction with several Nups and nuclear transport receptors on the cytoplasmic side of the nuclear pore (such as Nup88, Nup214, Nup98-RAE1, importin-β1 or RanBP2/Nup358) as well as on the nuclear pore lumen (as Nup54, Nup58, Nup93). SARS-CoV-2 infection also alters the shuttling of CRM1 exportin. For example, ORF6 inhibits IRF3 activation and STAT1 nuclear translocation. ORF6 interacts with Nup98-RAE1 as well as RanBP2/Nup358, Nup160, Nup 188, Nup210, Nup 37, Nup93, importins and exportin such as CRM1 and XPO3. As ORF6 interacts with nuclear pore complex proteins such as NUP98 and RAE1, the authors hypothesize that this may be a way to avoid nuclear translocations [39]. Another good example is Nsp1. Nsp1 has been observed near the nuclear pore complexes (NPCs) and directly binds the mRNA export factor NXF1, decreasing the availability of host cell mRNAs for the translation machinery, thus favoring viral mRNAs translation. For this reason, the overall transcriptome profile is altered by Nsp1 in infected cells [26]. A third example is the 98-aminoacids SARS-CoV-2 accessory protein 9b, small enough to enter the nucleus by passive transport. Furthermore, 9b interacts with CRM1 and gets exported out of the nucleus using an active NES. NES activity influences the half-life of 9b, and blocking the nuclear export of 9b induces apoptosis [37]. Interestingly, Ivermectin, which binds to and destabilizes nuclear importin Imp/1 heterodimer, prevents the suppression of antiviral response reducing viral load by ~5000 folds [19].

To sum up, certain SARS-CoV-2 proteins can hamper nucleocytoplasmic shuttling affecting nuclear export (e.g., affecting host mRNAs) or nuclear import (e.g., of host transcription factors involved in the antiviral response) [19]. Avoiding nuclear entry of a host cell transcription factor can be easily done from the cytoplasmic side of the nuclear pore. Affecting nuclear export requires a further step. Either the export is blocked through cytoplasmic-side sequestration/degradation of transport proteins such as CRM1 (which are otherwise constantly exiting the nucleus and then being recycled, entering the nucleus again) or—maybe more parsimoniously—the export is blocked through obstruction from the nuclear side of the pore (which requires nuclear entry of viral proteins). Do some viral proteins enter the nucleus?

### 3.3. SARS-CoV-2 Proteins: Nuclear Localization

Most viruses with ss(+) RNA genomes undergo replication in the cytoplasm, but some of their structural proteins localize to the nucleus, possibly inhibiting the host antiviral response [44]. N protein of some RNA viruses localizes to the nucleus/nucleolus of some infected cells. The coronavirus N protein is abundantly produced within infected cells and is one of the first clearly recognized as a Multifunctional Protein. CoV N proteins can localize to the host cell cytoplasm alone or to both the cytoplasm and the nucleus/nucleolus. Protein N has multiple roles, including virus replication, transcription, translation and ribonucleocapsid formation [45,46,47]. In host cells, N proteins have been shown to induce cell-cycle deregulation, inhibit the production of interferon, up-regulate the production of COX2 and up-regulate the activity of AP1. N interacts with numerous host cell proteins, including hCypA, proteasome subunit p42, the B23 phosphoprotein, Smad3, nRNP-A1, the chemokine CXCL16, translation elongation factor-1 alpha, cellular pyruvate kinase, 14-3-3 and nucleolin [38,48,49]. Nuclear translocation of the Nucleoprotein (N) has been demonstrated in several coronaviruses but has not been reported in SARS-CoV-2.

**Figure 3:**
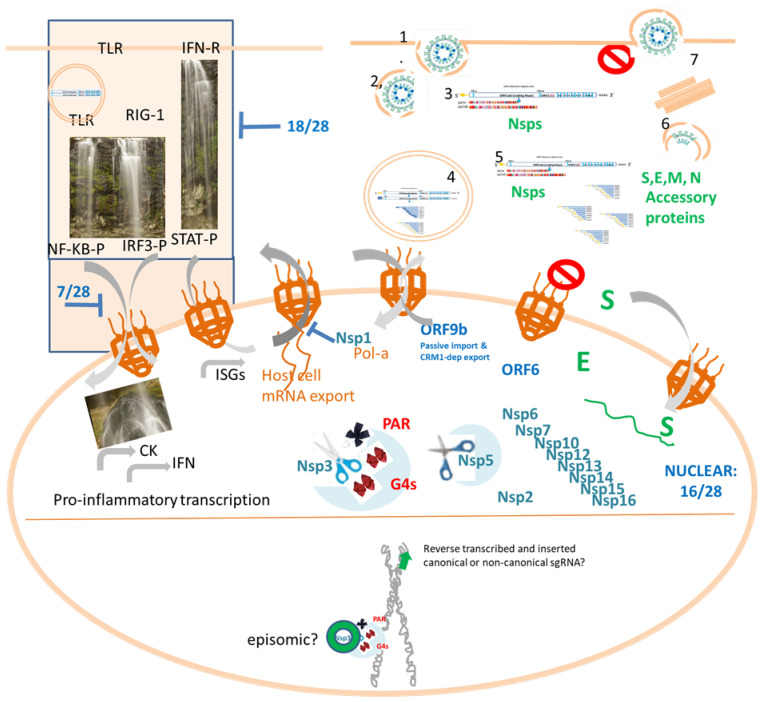
SARS-CoV-2 biology highlighting the importance of further studies of the interaction of canonical and non-canonical RNAs and proteins with host cell nucleus. *Top.* Canonical SARS-CoV-2 productive cycle (right) and IFN signaling interference (left). Proinflammatory signaling cascades involved in Cytokine (CK) and interferon (IFN) production, as well as IFN-dependent transcription, are hijacked by 18/28 SARS-CoV2 proteins upstream or at the transcription factor phosphorylation step while 7/28 can block the respective nucleocytoplasmic shutting of phosphorylated transctiption factors (NF-KB-P, IRF3-P or STAT-P). Bottom. The cell nucleus is represented displaying the SARS-CoV-2 proteins that have been detected in the nucleus, which constitute 16/28 proteins. Besides, a chromosome cartoon remembers us the episomic hypothesis to achieve persistence and the fact that at least under L1 overexpression, retrotranscribed copies of SARS-CoV2 sgRNAs is claimed to be inserted in the human genome.

Several viral proteins contain NLS and/or NES and localize to the nucleus (Figure 3). Unsurprisingly, SARS-CoV-2 Nsp1, involved in host mRNA export blocking, can reach the nucleus and interacts with DNA polymerase alpha (Pol a), an essential complex involved in DNA replication that couples cycle cell progression to DRR [27]. Nsp1 protein of SARS-CoV-2 prevents nuclear export of host mRNAs dependent on the receptor heterodimer NXF1-NXT1 through the interference with binding of NXF1 to mRNA export adaptors and NXF1 docking at the nuclear pore complex. NXF1 overexpression reverts Nsp1-mediated mRNA export block and reduction in mRNA levels. Although most Nsp1 is cytoplasmic, it does also colocalize with a nucleoporin (Nup358) and is even detected by ICF and confocal microscopy inside the nucleus near the nuclear envelope [24].

The SARS-CoV-2 surface glycoprotein Spike, coded by ORF2, presents five special features. First, it is optimized for human Angiotensin-converting enzyme 2 receptor (hACE2) binding [50]. Second, the Spike bears 4 HIV-like inserts with a high-density positive charge, very similar to HIV-1 surface proteins gp120 and Gag (as stated by Pradhan and Zhang in papers that were retracted for other reasons [51,52]), absent in other coronaviruses (absent even in SARS). For this reason, SARS-CoV-2 Spike, similar to HIV, binds CLEC4M (or CD299) and DC-SIGNR (or CD209), facilitating the infection of the immune system [53]. T-cells suffer exhaustion, and counts of total T cells are negatively correlated with patient survival [54]. Third, one of the insertions creates a furin-cleavable polybasic cleavage site (“PRRAR”). As both ACE2 receptors and furin protease are ubiquitous, this facilitates viral spreading across human tissues (and probably also across species) [6,55]. Fourth, the same insertion creates a superantigen (SAg) motif, namely CASYQTQT_NSPRRARSVASQSI, which was mapped due to its sequence similarity to the classical Sag called *Staphylococcus* enterotoxin B (SEB). Superantigens (SAgs) are a class of antigens produced by some pathogenic microbes as a defense mechanism against the host immune system. SAg cause the non-specific polyclonal activation of T-cells, massive cytokine release and hyperactivation of the immune system, which may lead to autoimmunity, multiple organ failure, and even death. The binding of this SAg to the T-cell receptor (TCR) may trigger the cytotoxic adaptive immune responses observed in multi-system inflammatory syndrome in children (MIS-C) as well as cytokine storms in adults with SARS-CoV-2 infection [31]. Finally, the same insertion creates a nuclear localization signal (NLS, specifically “PRRARSV”) overlapped with the polybasic cleavage site. Although Spike (S) is a glycoprotein, unlike in other coronaviruses, in the special case of SARS-CoV-2, Spike can reach the nucleus. In fact, if a highly differentiated pseudostratified airway epithelium is exposed to infection with SARS-CoV-2 (MOI: 0.1), fixed 4 days later and subject to immunocytofluorescence, 10% Spike protein is detected inside the host cell nucleus. In turn, 15% S protein is on the nuclear surface, and the remnant is cytoplasmic or membrane-bound. Consistently, 72 h after transfection of A549 cells with SARS-CoV-2 plasmid, SARS-CoV-2 protein is detected in cell homogenates in both cytoplasmic (CDC42+) and nuclear (Lamin A/C+) fractions [29,30].

The detection of nuclear Spike protein is a game changer, providing a new paradigm by which a direct effect on transcription by nuclear Spike protein would not be impossible, forcing us to re-interpret the experiments showing phenotypic changes in cells expressing ectopic Spike. Relevant experiments have been carried out. For example, the ectopic expression of Spike in cardiomyocytes derived from human induced pluripotent stem cells (hiPSCs) alters their metabolic profile and dampens their functions. Thus, the authors hypothesize that Spike can alter the transcriptional regulation in cardiac gene programs [32]. In the same line, SARS-CoV-2 infection of human lung cancer cell line transfected with ACE2 receptor (A549-ACE2 cells, MOI: 0.1) induced various pro-oncogenic signaling cascades including TGF-β signaling and epithelial to mesenchymal transition (EMT). As virus-induced metastasis has been found in many cancers, the authors focused on EMT mechanisms. In the MCF-7 breast cancer cell line, which has high ACE-2 expression [56], the ectopic expression of SARS-CoV-2 Spike, unlike other structural proteins (N, M, E), induces Snail-dependent (EMT), with E-cadherin downregulation, N-cadherin upregulation, increased migration and invasion [33]. Moreover, the ectopic expression of either SARS-CoV-2 Spike or SARS-CoV-2 Nucleoprotein is enough to induce lytic reactivation of Kaposi’s sarcoma-associated herpesvirus (KSHV), one of the major human oncogenic viruses in iSLK.219 cells [34].

In the extremely pathogenic SARS-CoV-2, SARS-CoV and highly related CoV found in bats but not other CoVs, Nsp3 protein (which harbors PL^PRO^ activity) contains a SARS Unique Domain (SUD) characterized by Macrodomains. Viral Macrodomains are considered unique mediators of viral replication and pathogenesis [57]. Canonical Macrodomains are “readers” of a post-translational protein modification called poly-ADP-ribosylation and also “erasers” of mono-ADP-ribosylation [58]. The crystal structure of the SARS-CoV macro domain was determined at 1.8-Å resolution in complex with ADP-ribose. Similar to other viral macro domains, from hepatitis E virus and Semliki Forest virus, it has poor ADP-ribose 1-phosphohydrolase activity but does efficiently bind poly (ADP-ribose) in vitro [59]. SARS-CoV Nsp3 SUD contains a canonical Macrodomain plus two atypical Macrodomains that bind G-quadruplexes (G4s) [60]; at least one of the G4-binding Macrodomains is essential for the activity of the SARS-CoV replication/transcription complex [61]. G4s are a particular structure of the nucleic acids that can arise in G-rich regions. Some viruses present G4s in their genome, while SARS-CoV could recognize G4s in host nucleic acids, for example, in 3′-nontranslated regions of mRNAs coding for host-cell proteins involved in apoptosis or signal transduction. G4s exist in the human genome, especially in telomeres and oncogene promoters [62]. In other viral infections, the recognition of G4s by viral proteins is involved in latency [63].

According to a systemic approach to reveal the subcellular locations of SARS-CoV-2 FLAGged proteins transfected in HEp-2 cells, some proteins were detected just in the cytoplasm, but the following ones were also present in the nucleus: NSP1, NSP3N, NSP5, NSP6, NSP7, NSP9, NSP10, NSP12, NSP13, NSP14, NSP15, NSP16, E and ORF9a [23]. Nsp13 is enriched in the splicing compartment. ORF3d has also been detected in the nucleus [19]. Many of these proteins are small and probably enter the nucleus by passive diffusion. NES may promote the nuclear export of these proteins or allow them to interact with exportins to hijack nuclear transportation. Anyway, pharmacological inhibition of nuclear export leads to nuclear accumulation of viral proteins and significantly decreases viral infection. We do absolutely agree with Chen et al., who state that “nuclear biology in understudied in COVID-19” [64]. Interestingly, ORF6, which is postulated to inhibit IFN-β transcription by altering nuclear translocation of STAT1 and IRF-3, has been detected under confocal ICF by Lei et al. (see Table 1, Table 2 and Table 3 and Figure 3) located predominantly in the cytoplasm, Golgi apparatus and endoplasmic reticulum, but also, to a lesser extent, inside the nucleus [39]

### 3.4. SARS-CoV-2 RNA: Nuclear Localization

After SARS-CoV-2 infection, not only SARS-CoV-2 Spike protein but also Spike mRNA is detected in the host cell nucleus. Moreover, Spike mRNA protein and mRNA exhibit certain colocalization. Nuclear translocation of Spike mRNA and protein is undoubtedly a novel feature of SARS-CoV-2 biology [30].

Single-molecule fluorescence in situ hybridization (smFISH) has allowed the detection of SARS-CoV-2 +gRNA, +sgRNA, –gRNA and dsRNA. Maximum z-projections of confocal images were required to quantify the RNA dispersion index of cytoplasmic signals (excluding DAPI-positive ROIs) on [65,66]. Some of the images suggest that it would be worth doing an analysis on successive single confocal images to reveal whether the few signals that colocalize with DAPI on maximum intensity z-projections should be interpreted as intranuclear or supranuclear (cytoplasmic, too) signals [65].

### 3.5. Evidence of SARS-CoV-2 sgRNA Retrotranscription and Insertion in the Host Genome

Chimeric viral-host reads in RNA-seq represent around 0.004–0.14% of the total SARS-CoV-2 reads in human samples across published datasets [67]. However, as RNA-seq library preparation is inherently error prone due to random template switching during reverse transcription of RNA to cDNA (with up to 1% of RNA-seq chimeric reads putatively artefactual) [68], the identification of genuine chimeric viral–cellular RNA transcripts is compromised by the generation of artifactual chimeras [67,68]. As retrotranscription and viral genomic integration, if happening, would be a low-frequency event, an experiment was designed by Zhang et al. using human Long Interspersed Nuclear Elements (LINEs) to increase the likelihood of detection of such events. LINEs are an endogenous cellular source of retrotranscriptase (RT) and are commonly over-expressed upon viral infection. LINEs are able to retrotranspose themselves and other non-autonomous elements, thus facilitating the integration in vertebrate genomes of DNA copies of non-retroviral RNA viruses. Therefore, HEK293T cells were transfected with LINE1 expression plasmids prior to infection with SARS-CoV-2. Then, genomic DNA was isolated from those cells 2 days after infection. DNA sequencing revealed target site duplications flanking the viral sequences and consensus LINE1 endonuclease recognition sequences at the integration sites, consistent with a LINE1 retrotransposon-mediated, target-primed reverse transcription and retroposition mechanism. Thus, SARS-CoV-2 sequences can be reverse-transcribed and integrated into the DNA of infected human cells in culture. Although infectious viruses cannot be produced from the integrated sub-genomic SARS-CoV-2 sequences, transcription of the integrated DNA copies could be responsible for positive PCR tests long after the initial infection. As a reference, only 1 in 1000 to 1 in 100,000 mouse cells infected with LCMV in culture or in vivo carried viral DNA copies integrated into the genome. Thus, if true, only a small fraction of cells in any patient tissues would be expected to be positive for viral sequences. No matter how challenging it may be, the authors propose “it will be important, in follow-up studies, to demonstrate the presence of SARS-CoV-2 sequences integrated into the host genome in patient tissues” [67].

**Figure 4:**
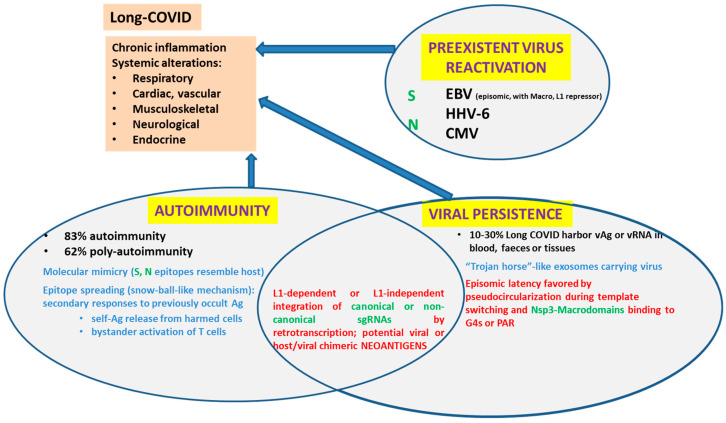
Main current hypotheses to explain Long-COVID and their relation to the direct or indirect interaction of SARS-CoV-2 proteins or RNAs with the host cell nucleus.

Smits et al. pose some reasonable doubts regarding Zhang et al.’s work related to SARS-CoV-2 insertion length, abundance and structure. In fact, Smits et al. [69] have tried to replicate Zhang et al. study, using the same cell line (HEK293T) relying on endogenous LINEs with no exit. Although the authors recognize that widespread cell death post-infection reduces the probability of SARS-CoV-2 integration persistence and recovery, they use a relatively high viral load for HEK293T (MOI:1). (As a reference, Zhang used 0.5 for HEK293T cells and Mehedi used MOI:0.1 in airway epithelium). Smits et al. argue they have a positive control which works as expected. The positive control is an HBV-positive cancer tissue, and they recovered a single HBV insertion [69]. HBV has been studied for a long while. It is known that cumulative HBV integrations in the human genome disrupt regulatory genes, drive aberrant gene expression and induce genomic rearrangements. For these reasons, HBV favors oncogenic transformation. According to Podlaha et al., although it is still unknown the proportion of hepatocytes that carry viral integrations, the HBV virus integrates with a lower-bound frequency of 0.84 per diploid genome in hepatitis B positive hepatocellular cancer patients, and it is calculated that integrated viral DNA generates ~80% of the HBsAg transcripts in these patients. Such viral or viral-host chimeric antigens may be driving chronic inflammation and/or autoimmunity [70]. If just a single HBV insertion was identified in the positive control, it does not seem strange not having been able to find small SARS-CoV-2 insertions corresponding to canonical or non-canonical sgRNAs retrotranscription. Thus, further studies are required to reach an agreement and solve this extremely relevant issue, represented with question marks in Figure 4 bottom nuclear region.

## 4. Current Hypotheses to Explain Long COVID

SARS-CoV 2 structure, which is similar to other β-coronaviruses and to SARS, soon led to hypothesize that it could cause systemic (rather than just respiratory) infection, affect the liver or the nervous system, cause hemagglutination, etc. Following the analogy, it was also hypothesized that it could cause persistent, chronic or latent infection. Cumulative evidence indicates that these fears were well justified [17,71,72,73,74,75,76,77,78,79,80,81,82] and more. “SARS-CoV-2 genomic-RNA can persist for many weeks in the respiratory tract of some individuals clinically recovered from coronavirus infectious disease-19 (COVID-19), despite a lack of isolation of infectious virus” [83]. SARS-CoV-2 was soon detected in the human brain. In a Brazilian neuroscience group’s words: “From where we now stand, it seems possible that, as currently infected individuals age in the coming years and decades, the systemic and/or brain inflammatory response elicited by SARS-CoV-2 infection may trigger long-term mechanisms leading to a widespread increase in the incidence of neurological and neurodegenerative disorders” [84,85]. A pediatric multi-system inflammatory syndrome similar to Kawasaki disease or toxic syndrome is associated with the COVID-19 pandemic, fortunately as a rare occurrence. Such children display fever, less than half display dyspnea and more than half have rashes, abdominal pain, vomiting or diarrhea. This was reported in Italy, the UK, the USA and France [86]. WHO defined precise criteria and opened a database to be able to follow the cases worldwide.

By definition, while reinfection occurs by a viral clade different from the first episode, reactivation or relapse of the infection takes place after symptom resolution, whereas the virus is still present but is regarded as dormant and may become active again. Reinfection is rare, below 1%, but reactivation rates suggest it is quite common [87]. One suggested mechanism of persistence, called the Trojan horse, is the hiding of viral particles in exosomes [87].

Long COVID was once a hypothesis. Now it is clearly established that SARS-CoV-2 causes Long COVID in a significant percentage of individuals within a few months after acute infection. According to the WHO, the post-COVID-19 condition, commonly known as Long COVID, can affect anyone exposed to SARS-CoV-2, regardless of age or severity of original symptoms. It is defined as the continuation or development of new symptoms 3 months after the initial SARS-CoV-2 infection, with these symptoms lasting for at least 2 months with no other explanation (https://www.who.int/europe/news-room/fact-sheets/item/post-covid-19-condition#:~:text=It%20is%20defined%20as%20the,months%20with%20no%20other%20explanation, accessed on 20 April 2023). From 10 to 30% of the hundreds of millions of people who had acute COVID-19 progressed to Long COVID, with the CDC reporting that 19% of adults who had COVID-19 are still suffering from symptoms. Long COVID seems to be systemic, involving symptoms that affect respiratory, cardiac, vascular, gastrointestinal, musculoskeletal, neurological or endocrine tissues/systems. Some symptoms, such as persistent inflammation and immune dysregulation, are shared with myalgic encephalomyelitis/chronic fatigue syndrome (ME/CFS) [31].

Five main non-exclusive hypotheses, recently reviewed by Vodjani et al. [31], have been proposed to explain Long COVID disease. We have already stated the fact Spike protein could behave as a Superantigen. We will not delve further into gut microbiota disturbances. Thus, we will revise (i) viral persistence, (ii) reactivation of latent viruses and (iii) autoimmunity and briefly discuss their possible role in chronic inflammation and Long COVID (Figure 4).

### 4.1. Viral Persistence

We will take Vodjani et al.’s definition of viral persistence literally. “Under certain conditions, a viral invader may not be completely eliminated by the host’s immune system. The cessation of symptoms, non-detection of viral presence, and the development of immunity after infection does not necessarily mean that the virus has been eradicated from the host. Some viruses or their parts may remain hidden in tissues and could potencially be reactivated. This is viral persistence.” In fact, SARS-CoV-2 viral RNA or viral antigens were detected in blood, feces and tissue biopsies from 3 to several months after initial COVID diagnosis. [31]. Their definition of viral persistence includes the possibility that viral parts may remain and hide in the tissues. In this sense, the presence of DNA copies of sgRNAs integrated into the host genome would be a novel way of (partial) viral persistence. Anyway, the expression of viral-host chimeric proteins could create neo-antigens that would activate autoimmunity and promote a chronic pro-inflammatory state.

The knowledge that ss(+) RNA viruses can develop latent infections is relatively new. A review entitled “Virus Latency and the Impact on Plants” lists eleven ss(+) RNA viruses with mono, di, or tripartite RNA segmentation, which remain latent until their reactivation which can be evidenced by specific symptoms [88]. If an ss(+) RNA virus can do it in plants, why not in animals? Interestingly, SARS-CoV-2 G4-binding Macrodomains resemble G4-binding proteins used as a hook by viruses that maintain episomic latency. One known mechanism to maintain episomic latency (which, by definition, does not involve DNA viral sequence integration in the host genome) is through the anchorage of a circular viral nucleic acid to host mitotic chromosomes. To this end, a virus may code a protein with a G4s-binding domain. The protein acts as a hook to transport viral sequences bound to host chromosomes [63]. Figure 2 in Lieberman’s article illustrates a model of how host chromosomes can give a shuttle to Epstein–Barr virus genome (EBV) or Kaposi Sarcoma Herpesvirus (KSHV). Episomal genomes have also been identified during a latent infection for human papilloma virus (HPV), cytomegalovirus (CMV) and herpes simplex virus (HSV). Although a specific mechanism for ss(+) RNA viruses episomal latency has not been described, SARS-CoV-2 SUD Macrodomains would be well-suited as a G4s-binding hook, while the pseudo-circular RNA structures generated due to template switching are a remnant of those described in Lieberman’s report for other viruses. Although initially just cytoplasmic CoV RNA sequences had been detected by fluorescent in situ hybridization (FISH), we argued that it could be worth trying to follow SARS dsRNAs during infection using a dsRNA binding-dependent fluorescent complementation assay and confocal microscopy [89]. A recent study has detected all types of viral RNAs in SARS-CoV-2 infected cells through FISH [65] but has focused on the cytoplasmic side, leaving putative nuclear immunofluorescent signals out of the analysis.

### 4.2. Reactivation of Latent Viruses

Several studies have reported a strong correlation between the length and severity of COVID symptoms and the reactivation of latent herpesviruses (mononucleosis-related EBV, HHV-6, CMV). Insomnia, headaches, myalgia and confusion characteristic of Long COVID could be explained in this way [31], particularly in systemic responders (with increased plasma antiviral antibodies titers) [90]. The mechanisms leading to viral reactivation are worth studying. How does the ectopic expression of either SARS-CoV-2 Spike or SARS-CoV-2 Nucleoprotein induce lytic reactivation of Kaposi’s sarcoma-associated herpesvirus (KSHV), one of the major human oncogenic viruses in iSLK.219 cells? [34].

### 4.3. Viral-Induced Autoimmunity

Viral-induced autoimmunity can be caused through different mechanisms such as molecular mimicry, epitope spreading, and bystander activation of uninfected cells [91]. Following infection with SARS-CoV-2, certain patients developed classical autoimmune diseases such as cardiomyopathy, type 1 diabetes, rheumatoid arthritis, psoriatic arthritis, lupus, idiopathic inflammatory myopathies, systemic Guillain–Barré syndrome, thyroid autoimmunity or sclerosis [31]. Many researchers believe that severe SARS-CoV-2 infection can lead to new potentially pathogenic antibodies that may attack host tissues and cause harm. Four key pieces of evidence support considering SARS-CoV-2 as one of the “autoimmune viruses”: (1) SARS-CoV-2 Spike proteins and nucleoproteins mimic human autoantigens involved with autoimmune diseases; (2) monoclonal antibodies (mAbs) made against SARS-CoV-2 Spike proteins and nucleoproteins react with human autoantigens; (3) antibodies against human autoantigens react with SARS-CoV-2 Spike proteins and nucleoproteins; and (4) the sera of patients with COVID-19 have tested positive for autoantibodies made against human autoantigens known to cross-react with SARS-CoV-2 [31]. Autoimmunity was detected in 83% and poly-autoimmunity in 62% of patients with post-COVID syndrome.

## 5. Discussion

This work presents evidence from the literature that indicates that SAR-CoV-2 infection strikes the cell nucleus and its functions in order to control the host cell machinery. SARS-CoV-2 affects phosphorylation and/or nuclear import of specific transcription factors such as NF-KB, IFR3 and IFN-β—thus modulating the host immune response—and precludes nuclear export of host mRNAs. Both Spike protein and Spike mRNA are detected inside the nucleus of cells in infected airway epithelium. At least when overexpressed, several SARS-CoV-2 proteins reach the nucleus or the nucleolus. The study of their roles inside the nucleus may shed light on Long COVID biology. Noteworthy, retrotranscription of SARS-CoV-2 sgRNAs occurs at least in vitro in cells overexpressing LINEs, and it is imperative to study whether this occurs in vivo, particularly in Long COVID patients. If SARS-CoV-2 infection triggers LINEs overexpression, then the reactivation of latent viruses, viral persistence, and viral-induced autoimmunity could be part of the same phenomenon.

## Figures and Tables

**Table 1:** SARS-CoV-2 non-structural proteins IFN signaling, nucleocytoplasmic traffic and localization.

Protein	Blocks TF Activation	Blocks TF Translocation	Interacts with NTRs or Nups	Detected in Nucleus?	Highlights/Comments
Nsp1	True No: STAT-P [22]		NXF1-NXT1 [22]	Yes [23,24]	Reduces host mRNA export [25]: Alters host cell transcriptome [26]: Inhibits HDAC2 transport [22]: Interacts with DNA Pol a [27].
Nsp2			No [23]		
Nsp3	True No: NFKB-P No: IRF3-P No: STAT-P [22]		Yes (NSP3-Nt) No NSP3-Ct [23]		PL^PRO^ Protease. 3 Macrodomains. Canonical Macrodomain binds PAR. Non-canonical Macrodomains bind G4s.
Nsp4			GP210 [28]	No [23]	
Nsp5	True No: IRF3-P [22]	IRF3 [25]		Yes [23]	3-CL^PRO^, Main Protease.
Nsp6	No> IRF3-P; STAT-P [25]			Yes [23]	
Nsp7				Yes [23]	Suppresses IFN-α signaling [22].
Nsp8	True; No>IRF3-P [22]			No [23]	
Nsp9	True [22]		Nup54, Nup58,Nup 62, Nup 88, Nup214 [28] Nup 62 [22]	Yes [23]	
Nsp10	True [22]			Yes [23]	
Nsp11					
Nsp12	True [22]	IRF3 [25]		Yes [23]	RNA-dep RNA-pol.
Nsp13	No> NF-KB-P; IRF3-P; STAT-P [22,25]			Yes [23]	Colocalizing with SC35 [23].
Nsp14	True [22]	IRF3 [25]		Yes [23]	Exoribonuclease
Nsp15	True [22]	IRF3 [25]	NTF2 [28]	Yes [23]	RNA endonuclease
Nsp16	True [25]			Yes [23]	

**Table 2:** SARS-CoV-2 structural proteins IFN signaling, nucleocytoplasmic traffic and localization.

Protein	Blocks TF Activation	Blocks TF Translocation	Interacts with NTRs or Nups	Detected in Nucleus?	Highlights/Comments
ORF2. S				No [23] Yes [29,30]	Has an NLS [30]; Bears a Superantigen motif [31]; Alters cardiomyocyte metabolism and functions [32]; Induces pro-oncogenic cascades [33]; Induces KSHV reactivation [34]; Predicted NES [35].
ORF4.E				Yes [23]	
ORF5.M				No [23]	
ORF9a.N	No: IRF3-P; No: STAT-P [25]	IRF3 STAT1/2 [25]		No [23]	Biphasic effect on IFN signaling. Low N concentration diminishes it, while high N concentration enhances it and could participate in cytokine storms [28]. Involved in liquid–liquid demixing and IKK sequestration [36]. Localizes to nucleus and nucleolus in IBV and MHV CoV, but not in SARS-CoV (in spite of 3 putative NLS, NoLS and NES).

**Table 3:** SARS-CoV-2 accessory proteins IFN signaling, nucleocytoplasmic traffic and localization.

Protein	Blocks TF Activation	Blocks TF Translocation	Interacts with NTRs or Nups	Detected in Nucleus?	Highlights/Comments
ORF3a	No: STAT-P [25]			No [23]	
ORF3b	True [25]			No [23]	Immunodominant protein. Induces high levels of antibodies [25]. Predicted NES: IITLKKRWQLAL [35]
ORF6	True [25]	IRF3. [25] STAT1 through Imp-α1, Impβ1 linkage to ER. Nup-98-RAE1 [28]	Imp-α1, Impβ1 Nup-98-RAE1 RanBP2/Nup358, Nup160, Nup188, Nup210, Nup 37, Nup93, Imp-5, Imp-8, RanBP6, XPO3, CRM1 [28]	No. [23]	Alters host mRNA transport [28]; Bidirectional transport disruption.
ORF7a	STAT-P [25]			No [23]	
ORF7b	STAT-P [25]			No [23]	
ORF8		IRF3 [25]		No [23]	NFKB promoter inhibition?
ORF9b			Small enough to enter through passive diffusion. Interacts with CRM1 exportin [37].	No [23] Yes [37]	Predicted NES [35]; Has an NES [37]; Affect IFN signaling through TOM70 [25]; Triggers apoptosis if retained in the nucleus [37]; Alters cardiomyocytes [32].
ORF10				No [23]	Not essential [25]

## Data Availability

Not applicable.

## Supplementary Materials

The following supporting information can be downloaded at: https://www.mdpi.com/article/10.3390/pathogens12060806/s1.
